# Genetics Selection Evolution reviewer acknowledgement 2015

**DOI:** 10.1186/s12711-016-0195-z

**Published:** 2016-03-02

**Authors:** Didier Boichard, Jack Dekkers, Helene Hayes, Julius van der Werf

**Affiliations:** GABI, INRA, AgroParisTech, Université Paris-Saclay, 78350 Jouy-en-Josas, France; Department of Animal Science and Center for Integrated Animal Genomics, Iowa State University, Ames, IA 50011 USA; School of Environmental and Rural Science, University of New England, Armidale, NSW 2351 Australia

## Reviewer acknowledgement

The *Genetics Selection Evolution* editorial team would sincerely like to thank all of our reviewers who contributed to peer review for the journal in 2015.

**Herve Acloque**

France

**Tormod Ådnøy**

Norway

**Samuel E. Aggrey**

USA

**CarmenAmador**

UK

**Peter Amer**

New Zealand

**Marcel Amills**

Spain

**Osvaldo Anacleto**

UK

**Jesus Arango**

USA

**Alan Archibald**

UK

**Eric Barrey**

France

**David Bartel**

USA

**Bertrand Bed’hom**

France

**Jonathan Beever**

USA

**Camillo Berenos**

UK

**Mairead Bermingham**

UK

**Donagh Berry**

Ireland

**Piter Bijma**

The Netherlands

**Filippo Biscarini**

Italy

**Agustin Blasco**

Spain

**Loys Bodin**

France

**Sunduimijid Bolormaa**

Australia

**Lanie Bourgeois**

USA

**Aniek Bouwman**

The Netherlands

**Horst Brandt**

Germany

**A. J. (Bart) Buitenhuis**

Denmark

**Mario Calus**

The Netherlands

**Javier Cañon**

Spain

**Aurélien Capitan**

France

**Fernando Cardoso**

Brazil

**Nuno Carolino**

Portugal

**Roberto Carvalheiro**

Brazil

**Yizhou Chen**

Australia

**Hans Cheng**

USA

**Ole F. Christensen**

Denmark

**Elena Ciani**

Italy

**Samuel Adam Clark**

Australia

**Noelle Cockett**

USA

**Jonathan Codd**

UK

**Erin Connor**

USA

**Wouter Coppieters**

Belgium

**E. Gus Cothran**

USA

**Paola Crepaldi**

Italy

**Pascal Croiseau**

France

**Richard Crooijmans**

The Netherlands

**Jose Crossa**

Mexico

**Karen Crow**

USA

**Beatriz Cuyabano**

Denmark

**Hans Daetwyler**

USA

**Binyam Sime Dagnachew**

Norway

**William Davidson**

Canada

**Gerben De Jong**

The Netherlands

**Dirk-Jan De Koning**

Sweden

**Jared Decker**

USA

**Joseph Deep**

USA

**Olivier Demeure**

France

**Johann Detilleux**

Belgium

**Terry Dial**

United States Minor Outlying Islands

**Corrado Dimauro**

Italy

**Xiangdong Ding**

China

**Ottmar Distl**

Germany

**Dayna Dreger**

USA

**Tom Druet**

Belgium

**Vincent Ducrocq**

France

**Ian Dunn**

UK

**Susana Dunner**

Spain

**Christian Edel**

Germany

**Esther Ellen**

The Netherlands

**Jean Michel Elsen**

France

**Mauricio Elzo**

USA

**Malena Erbe**

Germany

**Frederic Farnir**

Belgium

**Maja Ferencakovic**

Croatia

**Jesús Fernández**

Spain

**Rohan Fernando**

USA

**W. Freddy Fikse**

Sweden

**Luca Fontanesi**

Italy

**Gustavo Gandini**

Italy

**Luis Alberto Garcia Cortes**

Spain

**Maria C. Garcia Gil**

Spain

**Jose-Luis García-Marín**

Spain

**Giustino Gaspa**

Italy

**Mathieu Gautier**

France

**Nicolas Gengler**

Belgium

**Helene Gilbert**

France

**Linus Girdland Flink**

UK

**Kay-Uwe Goetz**

Germany

**Cedric Gondro**

Australia

**Oscar Gonzalez-Recio**

Spain

**Gregor Gorjanc**

UK

**Felix Goyache**

Spain

**Birgit Gredler**

Switzerland

**Darren Griffin**

UK

**Martien Groenen**

The Netherlands

**Bernt Guldbrandtsen**

Denmark

**Ximing Guo**

USA

**Juan Pablo Gutierrez**

Spain

**Olivier Hanotte**

UK

**Bevin Harris**

New Zealand

**Rie Henriksen**

Sweden

**John Henshall**

Australia

**Juan Manual Herrero-Medrano**

The Netherlands

**William Hill**

UK

**Paul Hocking**

UK

**Ross Houston**

UK

**David Howard**

UK

**Jisca Huisman**

UK

**Heather Huson**

USA

**Noelia Ibañez-Escriche**

Spain

**Eveline Ibeagha-Awemu**

Canada

**Julien Jacquin**

France

**Luc Janss**

Denmark

**Anna Johansson**

Sweden

**R.K. Johnson**

USA

**Amélie Juanchich**

France

**Haja Kadarmideen**

Denmark

**Juha Kantanen**

Finland

**Anti Kause**

Finland

**John William Keele**

USA

**Mehar S. Khatkar**

Australia

**Brian Kinghorn**

Australia

**Pieter Knap**

Germany

**Naama Kopelman**

Israel

**Robert Kraus**

Germany

**Larry Kuehn**

USA

**Catherine Larzul**

France

**Fabienne Le Provost**

France

**Jun-Heon Lee**

Republic of Korea

**Andres Legarra**

France

**Christina Lehermeier**

Germany

**Gregoire Leroy**

France

**Lian Lian**

USA

**Yves Lignereux**

France

**Marie Lillehammer**

Norway

**Daniela Lino Lourenco**

USA

**George Liu**

USA

**Daniela Lourenco**

USA

**Nicolò Macciotta**

Italy

**Michael Macneal**

USA

**Daniel Maizon**

Argentina

**Christian Maltecca**

USA

**Esa Mantysaari**

Finland

**Cecile Massault**

Australia

**Joel Mcglothlin**

USA

**Kathryn Mcrae**

New Zealand

**Gábor Mészáros**

Austria

**Theo Meuwissen**

Norway

**Karin Meyer**

Australia

**Sandrine Mignon-Grasteau**

France

**Thomas Moen**

Norway

**Carole Moreno**

France

**Gota Morota**

USA

**Raphael Mrode**

UK

**Han Mulder**

The Netherlands

**Ezequiel Luis Nicolazzi**

Italy

**Motohide Nishio**

Japan

**Jorgen Odegard**

Norway

**Pablo Orozco-Terwengel**

UK

**Yuchun Pan**

China

**Hubert Pausch**

Germany

**Francisco Penagaricano**

USA

**Miguel Perez-Enciso**

Spain

**Jaime Perez-Sanchez**

Spain

**Danillo Pinhal**

Brazil

**Geoff Pollott**

UK

**Jennie Pryce**

Australia

**Deirdre Purfield**

Ireland

**Herman Raadsma**

Australia

**Alexander Rebl**

Germany

**Norbert Reinsch**

Germany

**Antonio Reverter-Gómez**

Australia

**Anne Ricard**

France

**Annie Robic**

France

**Bas Rodenburg**

The Netherlands

**Silvia Teresa Rodriguez Ramilo**

Spain

**Rainer Roehe**

UK

**Gary Rohrer**

USA

**Megan Rolf**

USA

**Sergio-Ivan Roman-Ponce**

Mexico

**Lars Rönnegård**

Sweden

**Sophie Rothammer**

Germany

**Suzanne Rowe**

New Zealand

**Madhi Saatchi**

USA

**Goutam Sahana**

Denmark

**Guillaume Salle**

France

**Juan Pablo Sanchez**

Spain

**Marie-Pierre Sanchez**

France

**Magali San Cristobal**

France

**María Saura**

Spain

**Flavio Schenkel**

Canada

**Robert Schnabel**

USA

**Chris Schoen**

Germany

**Dirkjan Schokker**

The Netherlands

**Anna Schönherz**

Denmark

**Chris Schrooten**

The Netherlands

**Mohammad Shariati**

Iran

**Takashi Shiina**

Japan

**Henner Simianer**

Germany

**Warren Snelling**

USA

**Tad Sonstegard**

USA

**Jennifer Spindel**

USA

**Juan Steibel**

USA

**Ismo Stranden**

Finland

**Chuanyu Sun**

USA

**Miika Jani Juhani Tapio**

Finland

**Marinus Te Pas**

The Netherlands

**Jens Tetens**

Germany

**Georg Gt Thaller**

Germany

**Bruce Tier**

Australia

**Francesco Tiezzi**

Italy

**Michèle Tixier-Boichard**

France

**Miguel Angel Toro**

Spain

**Christopher Tuggle**

USA

**Mario Graziano Usai**

Italy

**Yuri Tani Utsunomiya**

Brazil

**Bruno Valente**

USA

**Rianne Van Binsbergen**

The Netherlands

**Irene van den Berg**

Denmark

**Julius van der Werf**

Australia

**Jeremie Vandenplas**

The Netherlands

**Marc Vandeputte**

France

**Paul Vanraden**

USA

**Luis Varona**

Spain

**Roel Veerkamp**

The Netherlands

**Johanna Vilkki**

Finland

**Rafael Villa-Angulo**

Mexico

**Beatriz Villanueva**

Spain

**Zulma Vitezica**

France

**Patrik Waldmann**

Sweden

**Barbara Wallner**

Austria

**Bruce Walsh**

USA

**Lei Wang**

Denmark

**Joel Ira Weller**

Israel

**Robin Wellmann**

Germany

**Ian White**

UK

**Yvonne Wientjes**

The Netherlands

**George Wiggans**

USA

**Klaus Wimmers**

Germany

**Jack Windig**

The Netherlands

**Dörte Wittenburg**

Germany

**Anna Wolc**

USA

**David Yañez-Ruiz**

Spain

